# 3,5-Diamino-4*H*-1,2,4-triazol-1-ium (6-carb­oxy­pyridine-2-carboxyl­ato)(pyridine-2,6-dicarboxyl­ato)cuprate(II) trihydrate

**DOI:** 10.1107/S1600536811011147

**Published:** 2011-03-31

**Authors:** S. Yousuf, A. S. Johnson, S. A. Kazmi, O. E. Offiong, Hoong-Kun Fun

**Affiliations:** a H.E.J Research Institute of Chemistry, International Center for Chemical and Biological Sciences, University of Karachi, Karachi 75270, Pakistan; b Department of Pure and Applied Chemistry, University of Calabar, Calabar, PMB 1115, Nigeria; c X-ray Crystallography Unit, School of Physics, Universiti Sains Malaysia, 11800 USM, Penang, Malaysia

## Abstract

In the complex anion of the title compound, (C_2_H_6_N_5_)[Cu(C_7_H_4_NO_4_)(C_7_H_3_NO_4_)]·3H_2_O, the Cu^II^ atom is coordinated by tridentate 6-carb­oxy­pyridine-2-carboxyl­ate and pyridine-2,6-dicarboxyl­ate ligands and is surrounded by four O atoms in the equatorial plane and two N atoms in axial positions in a distorted octa­hedral geometry. In the crystal, the components are linked into a three dimensional network by O—H⋯O, N—H⋯O, N—H⋯N and C—H⋯O hydrogen bonds and a π–π inter­action with a centroid–centroid distance of 3.6080 (8) Å.

## Related literature

For general background to and applications of supra­molecular arrangements, see: Lehn (1995 ▶); Aghajani *et al.* (2009 ▶); Tshuva & Lippard (2004 ▶); Kuzelka *et al.* (2003 ▶). For crystal structures of related complexes, see: Aghabozorg *et al.* (2007 ▶); Ramos Silva *et al.* (2008 ▶); Wang *et al.* (2004 ▶); MacDonald *et al.* (2004 ▶). For the stability of the temperature controller used in the data collection, see: Cosier & Glazer (1986 ▶).
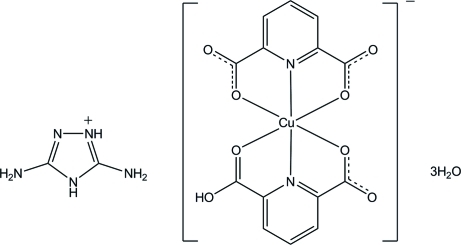


## Experimental

### 

#### Crystal data

(C_2_H_6_N_5_)[Cu(C_7_H_4_NO_4_)(C_7_H_3_NO_4_)]·3H_2_O
*M* *_r_* = 548.92
Orthorhombic, 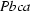

*a* = 11.3091 (2) Å
*b* = 14.9442 (3) Å
*c* = 24.6045 (5) Å
*V* = 4158.29 (14) Å^3^
*Z* = 8
Mo *K*α radiation
μ = 1.13 mm^−1^
*T* = 100 K
0.54 × 0.20 × 0.07 mm

#### Data collection

Bruker SMART APEXII DUO CCD area-detector diffractometer
Absorption correction: multi-scan (*SADABS*; Bruker, 2009 ▶) *T*_min_ = 0.583, *T*_max_ = 0.921
55434 measured reflections
9184 independent reflections
6619 reflections with *I* > 2σ(*I*)
*R*_int_ = 0.040

#### Refinement

*R*[*F* ^2^ > 2σ(*F* ^2^)] = 0.039
*wR*(*F* ^2^) = 0.098
*S* = 1.05
9184 reflections
368 parameters
H atoms treated by a mixture of independent and constrained refinement
Δρ_max_ = 0.61 e Å^−3^
Δρ_min_ = −0.62 e Å^−3^

### 

Data collection: *APEX2* (Bruker, 2009 ▶); cell refinement: *SAINT* (Bruker, 2009 ▶); data reduction: *SAINT*; program(s) used to solve structure: *SHELXTL* (Sheldrick, 2008 ▶); program(s) used to refine structure: *SHELXTL*; molecular graphics: *SHELXTL*; software used to prepare material for publication: *SHELXTL* and *PLATON* (Spek, 2009 ▶).

## Supplementary Material

Crystal structure: contains datablocks global, I. DOI: 10.1107/S1600536811011147/is2693sup1.cif

Structure factors: contains datablocks I. DOI: 10.1107/S1600536811011147/is2693Isup2.hkl

Additional supplementary materials:  crystallographic information; 3D view; checkCIF report

## Figures and Tables

**Table 1:** Hydrogen-bond geometry (Å, °)

*D*—H⋯*A*	*D*—H	H⋯*A*	*D*⋯*A*	*D*—H⋯*A*
O8—H1*O*8⋯O1^i^	0.90 (3)	1.71 (2)	2.5536 (15)	157 (2)
N3—H1*N*3⋯O2*W*^ii^	0.76 (2)	2.26 (2)	2.9081 (19)	144 (2)
N5—H1*N*5⋯O3*W*^iii^	0.80 (2)	2.23 (2)	2.8310 (17)	132.3 (18)
N5—H1*N*5⋯N4^iv^	0.80 (2)	2.40 (2)	2.9925 (18)	131.2 (19)
N6—H1*N*6⋯O2*W*^ii^	0.83 (2)	2.47 (2)	3.209 (2)	147.6 (19)
N6—H2*N*6⋯O3^v^	0.93 (2)	2.06 (2)	2.9699 (17)	168.7 (19)
N7—H1*N*7⋯O3*W*^iii^	0.81 (2)	2.15 (2)	2.8570 (19)	145 (2)
N7—H2*N*7⋯O7^vi^	0.82 (2)	1.96 (2)	2.7714 (18)	170 (2)
O1*W*—H1*W*1⋯O2	0.79 (2)	2.03 (3)	2.7985 (19)	168 (3)
O1*W*—H2*W*1⋯O5^v^	0.77 (3)	2.11 (3)	2.8715 (17)	171 (2)
O2*W*—H1*W*2⋯O1*W*	0.90 (3)	1.81 (3)	2.703 (2)	173 (3)
O2*W*—H2*W*2⋯O1^i^	0.85 (3)	2.58 (3)	3.3968 (18)	162 (3)
O3*W*—H1*W*3⋯O6^vii^	0.89 (3)	1.90 (3)	2.7712 (17)	169 (2)
O3*W*—H2*W*3⋯O3	0.77 (3)	2.04 (3)	2.8113 (16)	177 (2)
C5—H5*A*⋯O6^viii^	0.93	2.35	3.2042 (18)	153
C12—H12*A*⋯O7^i^	0.93	2.47	3.3960 (17)	176

Symmetry codes: (i) 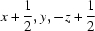; (ii) 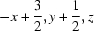; (iii) 
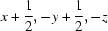
; (iv) 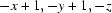; (v) 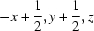; (vi) 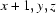; (vii) 
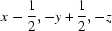
; (viii) 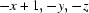.

**Fig. 1.**
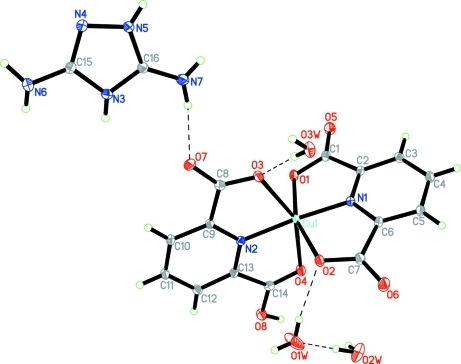
The molecular structure of the title crystal, showing 50% probability displacement ellipsoids and the atom-numbering scheme. The intramolecular hydrogen bonds are shown by dashed lines.

**Fig. 2.**
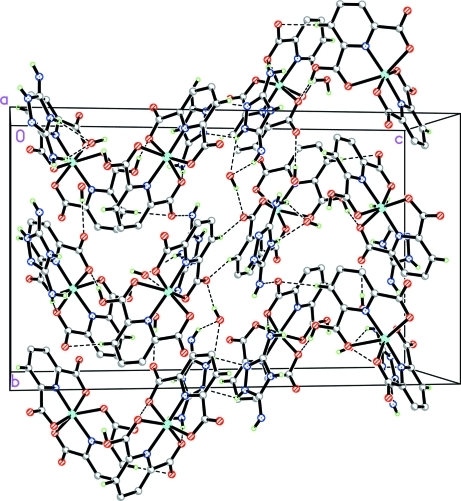
The crystal packing of the title compound, showing a three-dimensional molecular network. Only hydrogen atoms involved in hydrogen bonding are shown.

## supplementary crystallographic information

### Comment

Non-covalent supramolecular arrangements due to the non-covalent interactions between two or more molecular sub-units are responsible for influencing different structural properties and the addition of metal in the coordination is considered to be an effective tool to study and control the connectivity and linkages (Lehn *et al.*, 1995). The study of the construction of metal organic frameworks indicates that under suitable conditions, the transfer of the acidic protons to appropriate bases will result in increased intermolecular interactions and as a result enhanced stabilization of the resulting system (Aghajani *et al.*, 2009). The study of metal carboxylates has always been fascinating for the researchers because they play important roles not only in synthetic chemistry, considering the vast array of coordination modes of the carboxylate group, but also in biological activities (Tshuva *et al.*, 2004; Kuzelka *et al.*, 2003) and physiological effects (Aghabozorg *et al.*, 2007). In our ongoing research to study the packing features of molecules containing metal chelate and triazole rings, the title Cu complex (I) was prepared from 3,5-diamino-1,2,4-triazole and dipicolinic acid.

The title complex, (C_2_H_6_N_5_)^+^[Cu(C_7_H_4_NO_4_)(C_7_H_3_NO_4_)]^-^.3H_2_O, contains two tridentate dipicolinate ligands (one neutral and one protonated) coordinated with Cu(II) to reveal a distorted octahedral geometry, one independent protonated triazole and three water molecules (Fig. 1). The coordination environment around the Cu(II) ion is such that the two dipiconilate ligands are assembled perpendicular to each other with two axially oriented N atoms [Cu1—N1 = 1.9057 (12) Å, Cu1—N2 = 1.9723 (2) Å] and four O atoms on the basal positions [Cu1—O1 = 2.0889 (10) Å, Cu1—O2 = 2.0453 (10) Å, Cu1—O3 = 2.2727 (10) Å, Cu1—O4 = 2.3939 (10) Å]. Coordination of Cu^II^ ion with N1, O1, O2 and N2, O3, O4 of the two chelating dipicolinate ligands is responsible for the formation of four five-membered rings, A, (Cu1/O1/C1/C2/N1, with a maximum deviation of 0.049 (1) Å for atom O1), B (Cu1/N1/C6/C7/O2, with a maximum deviation of -0.042 (1) Å for atom O2), C (Cu1/O3/C8/C9/N2, with a maximum deviation of -0.043 (1) Å for atom C8) and D (Cu1/N2/C13/C14/O4, with a maximum deviation of -0.040 (1) Å for atom N2). The dihedral angles between them are 4.34 (6)° (A/B), 82.15 (6)° (A/C), 84.95 (6)° (A/D), 79.10 (6)° (B/C), 81.79 (6)° (B/D) and 3.58 (5)° (C/D). The independent triazole ring (N3/C15/N4/N5/C16) is essentially planar. All bond lengths are in agreement with another related structure (Ramos Silva *et al.*, 2008). O—H···O, N—H···O, N—H···N and C—H···O hydrogen bonds play important roles in stabilizing the crystal structure by forming a two-dimensional-network, which is further extended to three-dimensional-network due to the intermolecular linkages made by water solvates (Table 2 and Fig. 2). The three-dimensional network is further strengthened by significant π–π interactions between (N1/C1–C5) pyridine (centroid *Cg*5) and triazole (N3/C15/N4/N5/C16) (centroid *Cg*7) rings [*Cg*5···*Cg*7^vii^ distance = 3.6080 (8) Å; (vii) -1/2 + *x*, 1/2 - y, -*z*].

### Experimental

Pyridine-2,6-dicarboxylic acid (dipicolinic acid, H2dipic) and 3,5-diamino-1,2,4-triazole (datrz) were purchased from Merck and Molekula, respectively. Copper (II) sulfate pentahydrate (CuSO_4_.5H_2_O) and HPLC grade methanol were Uni- Chem and *M* TEDIA products, respectively. Deionized water was also used in the procedures when needed.

Synthesis of (Hdatrz)^+^[Cu(Hdipic)(dipic)]^-^.3H_2_O 1 mmol (0.099 g) of 3,5-diamino-1,2,4-triazole(datrz) and 1 mmol of dipicolinic acid (0.167 g) were dissolved in a mixture of methanol/water solution (1:10, 11 ml). The resulting solution was heated to 600 °C with stirring. An aqueous solution (1 ml) containing 0.5 mmol (0.125 g) of CuSO_4_.5H_2_O was added to the stirred solution. The greenish suspension was allowed to stir further for 1 hr, and then filtered while hot. The filtrate was kept at room temperature. Well shaped blue crystals of the title compound were formed by slow evaporation of the solution after 5 days. Percentage yield based on copper is 51.67%.

### Refinement

H atoms on C atoms were positioned geometrically, with C—H = 0.93 Å and constrained to ride, with *U*_iso_(H) = 1.2*U*_eq_(C). The H atoms on the oxygen and nitrogen atoms were located in a difference Fourier maps and refined isotropically; refined distances are O—H = 0.78 (3)–0.91 (3) Å and N—H = 0.76 (2)–0.92 (2) Å.

### Crystal data

**Table:**

(C_2_H_6_N_5_)[Cu(C_7_H_4_NO_4_)(C_7_H_3_NO_4_)]·3H_2_O	*D*_x_ = 1.754 Mg m^−^^3^
*M**_r_* = 548.92	Mo *K*α radiation, λ = 0.71073 Å
Orthorhombic, *P**b**c**a*	Cell parameters from 9982 reflections
*a* = 11.3091 (2) Å	θ = 2.4–34.3°
*b* = 14.9442 (3) Å	µ = 1.13 mm^−^^1^
*c* = 24.6045 (5) Å	*T* = 100 K
*V* = 4158.29 (14) Å^3^	Block, blue
*Z* = 8	0.54 × 0.20 × 0.07 mm
*F*(000) = 2248	

### Data collection

**Table:**

Bruker SMART APEXII DUO CCD area-detector diffractometer	9184 independent reflections
Radiation source: fine-focus sealed tube	6619 reflections with *I* > 2σ(*I*)
graphite	*R*_int_ = 0.040
φ and ω scans	θ_max_ = 35.2°, θ_min_ = 2.4°
Absorption correction: multi-scan (*SADABS*; Bruker, 2009)	*h* = −16→18
*T*_min_ = 0.583, *T*_max_ = 0.921	*k* = −24→24
55434 measured reflections	*l* = −35→39

### Refinement

**Table:**

Refinement on *F*^2^	Primary atom site location: structure-invariant direct methods
Least-squares matrix: full	Secondary atom site location: difference Fourier map
*R*[*F*^2^ > 2σ(*F*^2^)] = 0.039	Hydrogen site location: inferred from neighbouring sites
*wR*(*F*^2^) = 0.098	H atoms treated by a mixture of independent and constrained refinement
*S* = 1.05	*w* = 1/[σ^2^(*F*_o_^2^) + (0.0419*P*)^2^ + 1.6423*P*] where *P* = (*F*_o_^2^ + 2*F*_c_^2^)/3
9184 reflections	(Δ/σ)_max_ = 0.001
368 parameters	Δρ_max_ = 0.61 e Å^−^^3^
0 restraints	Δρ_min_ = −0.62 e Å^−^^3^

### Special details

**Table:**

Experimental. The crystal was placed in the cold stream of an Oxford Cryosystems Cobra open-flow nitrogen cryostat (Cosier & Glazer, 1986) operating at 100.0 (1) K.
Geometry. All e.s.d.'s (except the e.s.d. in the dihedral angle between two l.s. planes) are estimated using the full covariance matrix. The cell e.s.d.'s are taken into account individually in the estimation of e.s.d.'s in distances, angles and torsion angles; correlations between e.s.d.'s in cell parameters are only used when they are defined by crystal symmetry. An approximate (isotropic) treatment of cell e.s.d.'s is used for estimating e.s.d.'s involving l.s. planes.
Refinement. Refinement of *F*^2^ against ALL reflections. The weighted *R*-factor *wR* and goodness of fit *S* are based on *F*^2^, conventional *R*-factors *R* are based on *F*, with *F* set to zero for negative *F*^2^. The threshold expression of *F*^2^ > σ(*F*^2^) is used only for calculating *R*-factors(gt) *etc*. and is not relevant to the choice of reflections for refinement. *R*-factors based on *F*^2^ are statistically about twice as large as those based on *F*, and *R*- factors based on ALL data will be even larger.

### Fractional atomic coordinates and isotropic or equivalent isotropic displacement parameters (Å^2^)

**Table:**

	*x*	*y*	*z*	*U*_iso_*/*U*_eq_	
Cu1	0.215732 (15)	0.157389 (11)	0.147781 (7)	0.00961 (5)	
O1	0.08015 (9)	0.08189 (7)	0.18273 (4)	0.0134 (2)	
O2	0.36032 (9)	0.18850 (7)	0.10156 (4)	0.01296 (19)	
O3	0.07633 (9)	0.24435 (7)	0.10732 (4)	0.01269 (19)	
O4	0.35335 (9)	0.12522 (7)	0.21908 (4)	0.0141 (2)	
O5	−0.02108 (10)	−0.04545 (7)	0.16838 (5)	0.0178 (2)	
O6	0.49003 (10)	0.12228 (8)	0.04550 (5)	0.0209 (2)	
O7	−0.02109 (9)	0.37240 (7)	0.12527 (4)	0.0146 (2)	
O8	0.43168 (10)	0.20192 (7)	0.28899 (4)	0.0153 (2)	
N1	0.22886 (10)	0.04926 (8)	0.10717 (5)	0.0101 (2)	
N2	0.21368 (10)	0.26548 (8)	0.19377 (5)	0.0093 (2)	
N3	0.74524 (12)	0.52025 (8)	0.08876 (5)	0.0135 (2)	
N4	0.57456 (11)	0.54813 (8)	0.04709 (5)	0.0132 (2)	
N5	0.61177 (11)	0.45979 (8)	0.03913 (5)	0.0131 (2)	
N6	0.66479 (13)	0.66858 (9)	0.09430 (6)	0.0173 (3)	
N7	0.77259 (13)	0.36686 (10)	0.06458 (7)	0.0211 (3)	
C1	0.06033 (13)	0.00591 (9)	0.15837 (6)	0.0122 (2)	
C2	0.14997 (12)	−0.01609 (9)	0.11494 (6)	0.0102 (2)	
C3	0.15723 (12)	−0.09465 (9)	0.08511 (6)	0.0120 (2)	
H3A	0.1014	−0.1397	0.0895	0.014*	
C4	0.25034 (13)	−0.10441 (9)	0.04841 (6)	0.0126 (3)	
H4A	0.2568	−0.1565	0.0280	0.015*	
C5	0.33380 (12)	−0.03646 (9)	0.04217 (6)	0.0125 (3)	
H5A	0.3973	−0.0430	0.0185	0.015*	
C6	0.31922 (12)	0.04136 (9)	0.07243 (6)	0.0110 (2)	
C7	0.39802 (13)	0.12317 (10)	0.07216 (6)	0.0128 (3)	
C8	0.05761 (12)	0.31610 (9)	0.13374 (6)	0.0109 (2)	
C9	0.14039 (12)	0.33297 (9)	0.18110 (6)	0.0096 (2)	
C10	0.14068 (12)	0.41254 (9)	0.21033 (6)	0.0121 (2)	
H10A	0.0887	0.4585	0.2013	0.015*	
C11	0.21941 (12)	0.42253 (9)	0.25307 (6)	0.0127 (2)	
H11A	0.2219	0.4757	0.2727	0.015*	
C12	0.29478 (12)	0.35219 (9)	0.26639 (6)	0.0122 (2)	
H12A	0.3481	0.3574	0.2950	0.015*	
C13	0.28853 (12)	0.27413 (9)	0.23597 (6)	0.0108 (2)	
C14	0.36167 (12)	0.19255 (9)	0.24695 (6)	0.0116 (2)	
C15	0.65778 (12)	0.58205 (9)	0.07748 (6)	0.0123 (3)	
C16	0.71346 (12)	0.44283 (10)	0.06413 (6)	0.0125 (2)	
O1W	0.54413 (15)	0.26343 (9)	0.16261 (7)	0.0380 (4)	
O2W	0.64627 (12)	0.10349 (10)	0.18324 (6)	0.0293 (3)	
O3W	0.08779 (10)	0.22067 (8)	−0.00591 (5)	0.0171 (2)	
H1O8	0.467 (2)	0.1503 (17)	0.2979 (11)	0.054 (8)*	
H1N3	0.7969 (19)	0.5288 (15)	0.1077 (9)	0.032 (6)*	
H1N5	0.5722 (17)	0.4258 (14)	0.0218 (9)	0.023 (5)*	
H1N6	0.7083 (18)	0.6739 (15)	0.1215 (10)	0.028 (6)*	
H2N6	0.5929 (19)	0.6983 (14)	0.0952 (8)	0.026 (5)*	
H1N7	0.7412 (19)	0.3244 (14)	0.0499 (8)	0.022 (5)*	
H2N7	0.837 (2)	0.3643 (15)	0.0799 (9)	0.033 (6)*	
H1W1	0.491 (2)	0.2500 (19)	0.1436 (11)	0.049 (8)*	
H2W1	0.546 (2)	0.3146 (17)	0.1640 (10)	0.031 (6)*	
H1W2	0.611 (3)	0.157 (2)	0.1794 (14)	0.076 (10)*	
H2W2	0.618 (3)	0.0882 (19)	0.2141 (13)	0.067 (9)*	
H1W3	0.054 (2)	0.2667 (17)	−0.0225 (10)	0.044 (7)*	
H2W3	0.082 (2)	0.2265 (15)	0.0251 (11)	0.037 (7)*	

### Atomic displacement parameters (Å^2^)

**Table:**

	*U*^11^	*U*^22^	*U*^33^	*U*^12^	*U*^13^	*U*^23^
Cu1	0.01071 (8)	0.00748 (8)	0.01064 (8)	0.00011 (6)	0.00135 (6)	−0.00198 (6)
O1	0.0145 (5)	0.0100 (4)	0.0157 (5)	−0.0004 (4)	0.0043 (4)	−0.0022 (4)
O2	0.0146 (5)	0.0096 (4)	0.0147 (5)	−0.0009 (4)	0.0026 (4)	−0.0022 (4)
O3	0.0147 (5)	0.0113 (4)	0.0122 (5)	−0.0003 (4)	−0.0010 (4)	−0.0022 (4)
O4	0.0171 (5)	0.0100 (4)	0.0151 (5)	0.0020 (4)	−0.0017 (4)	−0.0012 (4)
O5	0.0169 (5)	0.0141 (5)	0.0223 (6)	−0.0037 (4)	0.0070 (4)	−0.0014 (4)
O6	0.0181 (5)	0.0179 (5)	0.0268 (6)	−0.0039 (4)	0.0116 (5)	−0.0058 (5)
O7	0.0134 (5)	0.0142 (5)	0.0163 (5)	0.0027 (4)	−0.0024 (4)	0.0013 (4)
O8	0.0183 (5)	0.0120 (5)	0.0157 (5)	0.0036 (4)	−0.0069 (4)	−0.0006 (4)
N1	0.0104 (5)	0.0095 (5)	0.0104 (5)	0.0004 (4)	0.0003 (4)	−0.0007 (4)
N2	0.0095 (5)	0.0088 (5)	0.0096 (5)	0.0003 (4)	0.0006 (4)	0.0002 (4)
N3	0.0120 (5)	0.0125 (5)	0.0160 (6)	−0.0003 (4)	−0.0038 (5)	−0.0010 (5)
N4	0.0125 (5)	0.0118 (5)	0.0155 (6)	0.0013 (4)	−0.0004 (4)	−0.0013 (4)
N5	0.0123 (5)	0.0108 (5)	0.0161 (6)	0.0001 (4)	−0.0026 (4)	−0.0020 (4)
N6	0.0174 (6)	0.0127 (6)	0.0217 (7)	0.0010 (5)	−0.0021 (5)	−0.0050 (5)
N7	0.0168 (7)	0.0120 (6)	0.0346 (8)	0.0021 (5)	−0.0115 (6)	−0.0028 (5)
C1	0.0141 (6)	0.0089 (6)	0.0136 (6)	0.0012 (5)	0.0017 (5)	0.0002 (5)
C2	0.0099 (6)	0.0090 (6)	0.0118 (6)	0.0008 (5)	0.0003 (4)	−0.0003 (4)
C3	0.0134 (6)	0.0089 (6)	0.0139 (6)	−0.0001 (5)	−0.0004 (5)	−0.0001 (5)
C4	0.0142 (6)	0.0109 (6)	0.0127 (6)	0.0019 (5)	−0.0017 (5)	−0.0017 (5)
C5	0.0126 (6)	0.0124 (6)	0.0125 (6)	0.0022 (5)	0.0008 (5)	−0.0021 (5)
C6	0.0115 (6)	0.0101 (6)	0.0113 (6)	0.0013 (5)	0.0009 (5)	−0.0005 (5)
C7	0.0143 (6)	0.0113 (6)	0.0128 (6)	−0.0008 (5)	0.0013 (5)	−0.0011 (5)
C8	0.0112 (6)	0.0110 (6)	0.0105 (6)	−0.0016 (5)	0.0007 (5)	0.0023 (5)
C9	0.0097 (5)	0.0086 (6)	0.0106 (6)	−0.0001 (4)	0.0007 (4)	−0.0001 (4)
C10	0.0122 (6)	0.0087 (6)	0.0155 (7)	0.0017 (5)	0.0005 (5)	−0.0010 (5)
C11	0.0141 (6)	0.0090 (6)	0.0150 (6)	0.0004 (5)	0.0008 (5)	−0.0026 (5)
C12	0.0134 (6)	0.0111 (6)	0.0122 (6)	−0.0001 (5)	−0.0018 (5)	−0.0018 (5)
C13	0.0108 (6)	0.0106 (6)	0.0108 (6)	0.0014 (5)	−0.0004 (5)	−0.0005 (4)
C14	0.0118 (6)	0.0116 (6)	0.0114 (6)	−0.0001 (5)	0.0003 (5)	0.0019 (5)
C15	0.0119 (6)	0.0116 (6)	0.0136 (6)	0.0010 (5)	0.0008 (5)	0.0003 (5)
C16	0.0116 (6)	0.0112 (6)	0.0146 (7)	−0.0005 (5)	−0.0020 (5)	0.0000 (5)
O1W	0.0469 (9)	0.0118 (6)	0.0554 (10)	−0.0030 (6)	−0.0334 (8)	0.0027 (6)
O2W	0.0308 (7)	0.0313 (7)	0.0256 (7)	0.0163 (6)	0.0061 (6)	0.0020 (6)
O3W	0.0232 (6)	0.0141 (5)	0.0140 (6)	0.0026 (4)	0.0017 (4)	−0.0017 (4)

### Geometric parameters (Å, °)

**Table:**

Cu1—N1	1.9057 (12)	N6—H2N6	0.93 (2)
Cu1—N2	1.9723 (12)	N7—C16	1.318 (2)
Cu1—O2	2.0453 (10)	N7—H1N7	0.81 (2)
Cu1—O1	2.0889 (10)	N7—H2N7	0.82 (2)
Cu1—O3	2.2727 (10)	C1—C2	1.509 (2)
Cu1—O4	2.3939 (10)	C2—C3	1.3870 (19)
O1—C1	1.3034 (17)	C3—C4	1.395 (2)
O2—C7	1.2877 (17)	C3—H3A	0.9300
O3—C8	1.2716 (17)	C4—C5	1.395 (2)
O4—C14	1.2213 (17)	C4—H4A	0.9300
O5—C1	1.2237 (17)	C5—C6	1.3907 (19)
O6—C7	1.2301 (17)	C5—H5A	0.9300
O7—C8	1.2425 (17)	C6—C7	1.513 (2)
O8—C14	1.3102 (17)	C8—C9	1.516 (2)
O8—H1O8	0.90 (3)	C9—C10	1.3896 (19)
N1—C2	1.3365 (17)	C10—C11	1.386 (2)
N1—C6	1.3375 (18)	C10—H10A	0.9300
N2—C9	1.3422 (17)	C11—C12	1.3925 (19)
N2—C13	1.3459 (17)	C11—H11A	0.9300
N3—C16	1.3547 (19)	C12—C13	1.3878 (19)
N3—C15	1.3813 (19)	C12—H12A	0.9300
N3—H1N3	0.76 (2)	C13—C14	1.4978 (19)
N4—C15	1.3045 (18)	O1W—H1W1	0.78 (3)
N4—N5	1.3994 (17)	O1W—H2W1	0.77 (2)
N5—C16	1.3285 (18)	O2W—H1W2	0.90 (3)
N5—H1N5	0.80 (2)	O2W—H2W2	0.85 (3)
N6—C15	1.3602 (19)	O3W—H1W3	0.89 (3)
N6—H1N6	0.83 (2)	O3W—H2W3	0.77 (3)
			
N1—Cu1—N2	174.99 (5)	C2—C3—C4	118.42 (13)
N1—Cu1—O2	80.73 (4)	C2—C3—H3A	120.8
N2—Cu1—O2	98.17 (4)	C4—C3—H3A	120.8
N1—Cu1—O1	79.34 (4)	C3—C4—C5	120.39 (13)
N2—Cu1—O1	101.40 (4)	C3—C4—H4A	119.8
O2—Cu1—O1	159.80 (4)	C5—C4—H4A	119.8
N1—Cu1—O3	108.01 (4)	C6—C5—C4	118.01 (13)
N2—Cu1—O3	76.99 (4)	C6—C5—H5A	121.0
O2—Cu1—O3	100.43 (4)	C4—C5—H5A	121.0
O1—Cu1—O3	88.85 (4)	N1—C6—C5	120.44 (13)
N1—Cu1—O4	99.40 (4)	N1—C6—C7	112.42 (12)
N2—Cu1—O4	75.63 (4)	C5—C6—C7	127.13 (13)
O2—Cu1—O4	86.18 (4)	O6—C7—O2	126.01 (14)
O1—Cu1—O4	93.85 (4)	O6—C7—C6	119.46 (13)
O3—Cu1—O4	152.47 (4)	O2—C7—C6	114.53 (12)
C1—O1—Cu1	114.05 (9)	O7—C8—O3	127.20 (13)
C7—O2—Cu1	113.87 (9)	O7—C8—C9	117.30 (13)
C8—O3—Cu1	111.94 (9)	O3—C8—C9	115.50 (12)
C14—O4—Cu1	107.22 (9)	N2—C9—C10	121.43 (13)
C14—O8—H1O8	111.8 (17)	N2—C9—C8	115.77 (12)
C2—N1—C6	122.47 (12)	C10—C9—C8	122.80 (12)
C2—N1—Cu1	119.51 (9)	C11—C10—C9	119.10 (13)
C6—N1—Cu1	118.00 (9)	C11—C10—H10A	120.4
C9—N2—C13	119.69 (12)	C9—C10—H10A	120.4
C9—N2—Cu1	119.31 (9)	C10—C11—C12	119.38 (13)
C13—N2—Cu1	120.92 (9)	C10—C11—H11A	120.3
C16—N3—C15	106.92 (12)	C12—C11—H11A	120.3
C16—N3—H1N3	128.6 (17)	C13—C12—C11	118.45 (13)
C15—N3—H1N3	124.2 (17)	C13—C12—H12A	120.8
C15—N4—N5	103.28 (11)	C11—C12—H12A	120.8
C16—N5—N4	112.06 (12)	N2—C13—C12	121.93 (12)
C16—N5—H1N5	127.6 (14)	N2—C13—C14	114.09 (12)
N4—N5—H1N5	120.4 (14)	C12—C13—C14	123.97 (12)
C15—N6—H1N6	111.6 (15)	O4—C14—O8	125.31 (13)
C15—N6—H2N6	114.3 (13)	O4—C14—C13	121.79 (13)
H1N6—N6—H2N6	117.0 (19)	O8—C14—C13	112.90 (12)
C16—N7—H1N7	116.6 (15)	N4—C15—N6	125.87 (13)
C16—N7—H2N7	119.6 (16)	N4—C15—N3	111.84 (13)
H1N7—N7—H2N7	124 (2)	N6—C15—N3	122.19 (13)
O5—C1—O1	125.68 (13)	N7—C16—N5	127.42 (14)
O5—C1—C2	120.75 (13)	N7—C16—N3	126.69 (13)
O1—C1—C2	113.57 (12)	N5—C16—N3	105.89 (13)
N1—C2—C3	120.22 (12)	H1W1—O1W—H2W1	107 (3)
N1—C2—C1	112.99 (12)	H1W2—O2W—H2W2	100 (3)
C3—C2—C1	126.79 (12)	H1W3—O3W—H2W3	109 (2)
			
N1—Cu1—O1—C1	−6.84 (10)	N1—C2—C3—C4	1.9 (2)
N2—Cu1—O1—C1	178.21 (10)	C1—C2—C3—C4	−176.87 (13)
O2—Cu1—O1—C1	−16.30 (18)	C2—C3—C4—C5	0.1 (2)
O3—Cu1—O1—C1	101.73 (10)	C3—C4—C5—C6	−1.6 (2)
O4—Cu1—O1—C1	−105.69 (10)	C2—N1—C6—C5	0.6 (2)
N1—Cu1—O2—C7	−5.71 (10)	Cu1—N1—C6—C5	179.32 (10)
N2—Cu1—O2—C7	169.34 (10)	C2—N1—C6—C7	−178.28 (12)
O1—Cu1—O2—C7	3.71 (18)	Cu1—N1—C6—C7	0.41 (15)
O3—Cu1—O2—C7	−112.48 (10)	C4—C5—C6—N1	1.3 (2)
O4—Cu1—O2—C7	94.47 (10)	C4—C5—C6—C7	−179.91 (14)
N1—Cu1—O3—C8	176.48 (9)	Cu1—O2—C7—O6	−171.91 (13)
N2—Cu1—O3—C8	−3.90 (9)	Cu1—O2—C7—C6	7.35 (15)
O2—Cu1—O3—C8	−99.97 (9)	N1—C6—C7—O6	173.99 (13)
O1—Cu1—O3—C8	98.08 (9)	C5—C6—C7—O6	−4.8 (2)
O4—Cu1—O3—C8	1.95 (14)	N1—C6—C7—O2	−5.33 (18)
N1—Cu1—O4—C14	174.94 (10)	C5—C6—C7—O2	175.84 (14)
N2—Cu1—O4—C14	−4.45 (9)	Cu1—O3—C8—O7	−172.04 (12)
O2—Cu1—O4—C14	95.00 (10)	Cu1—O3—C8—C9	7.09 (14)
O1—Cu1—O4—C14	−105.24 (9)	C13—N2—C9—C10	0.7 (2)
O3—Cu1—O4—C14	−10.33 (14)	Cu1—N2—C9—C10	−176.17 (10)
N2—Cu1—N1—C2	103.6 (6)	C13—N2—C9—C8	−178.69 (12)
O2—Cu1—N1—C2	−178.65 (11)	Cu1—N2—C9—C8	4.45 (16)
O1—Cu1—N1—C2	4.64 (10)	O7—C8—C9—N2	171.23 (12)
O3—Cu1—N1—C2	−80.62 (11)	O3—C8—C9—N2	−7.99 (18)
O4—Cu1—N1—C2	96.82 (10)	O7—C8—C9—C10	−8.1 (2)
N2—Cu1—N1—C6	−75.1 (6)	O3—C8—C9—C10	172.64 (13)
O2—Cu1—N1—C6	2.62 (10)	N2—C9—C10—C11	0.7 (2)
O1—Cu1—N1—C6	−174.09 (11)	C8—C9—C10—C11	−179.93 (13)
O3—Cu1—N1—C6	100.66 (10)	C9—C10—C11—C12	−1.2 (2)
O4—Cu1—N1—C6	−81.90 (10)	C10—C11—C12—C13	0.3 (2)
N1—Cu1—N2—C9	175.3 (5)	C9—N2—C13—C12	−1.7 (2)
O2—Cu1—N2—C9	98.28 (10)	Cu1—N2—C13—C12	175.13 (10)
O1—Cu1—N2—C9	−86.74 (10)	C9—N2—C13—C14	177.65 (12)
O3—Cu1—N2—C9	−0.61 (10)	Cu1—N2—C13—C14	−5.54 (16)
O4—Cu1—N2—C9	−177.83 (11)	C11—C12—C13—N2	1.2 (2)
N1—Cu1—N2—C13	−1.5 (6)	C11—C12—C13—C14	−178.06 (13)
O2—Cu1—N2—C13	−78.54 (11)	Cu1—O4—C14—O8	−177.73 (12)
O1—Cu1—N2—C13	96.45 (11)	Cu1—O4—C14—C13	3.18 (16)
O3—Cu1—N2—C13	−177.43 (11)	N2—C13—C14—O4	0.74 (19)
O4—Cu1—N2—C13	5.36 (10)	C12—C13—C14—O4	−179.95 (14)
C15—N4—N5—C16	0.02 (16)	N2—C13—C14—O8	−178.46 (12)
Cu1—O1—C1—O5	−172.99 (12)	C12—C13—C14—O8	0.9 (2)
Cu1—O1—C1—C2	7.53 (15)	N5—N4—C15—N6	176.70 (14)
C6—N1—C2—C3	−2.3 (2)	N5—N4—C15—N3	0.33 (16)
Cu1—N1—C2—C3	179.01 (10)	C16—N3—C15—N4	−0.57 (17)
C6—N1—C2—C1	176.63 (12)	C16—N3—C15—N6	−177.08 (14)
Cu1—N1—C2—C1	−2.03 (16)	N4—N5—C16—N7	−179.65 (15)
O5—C1—C2—N1	176.49 (13)	N4—N5—C16—N3	−0.36 (16)
O1—C1—C2—N1	−4.00 (17)	C15—N3—C16—N7	179.84 (15)
O5—C1—C2—C3	−4.6 (2)	C15—N3—C16—N5	0.54 (16)
O1—C1—C2—C3	174.87 (13)		

### Hydrogen-bond geometry (Å, °)

**Table:**

*D*—H···*A*	*D*—H	H···*A*	*D*···*A*	*D*—H···*A*
O8—H1O8···O1^i^	0.90 (3)	1.71 (2)	2.5536 (15)	157 (2)
N3—H1N3···O2W^ii^	0.76 (2)	2.26 (2)	2.9081 (19)	144 (2)
N5—H1N5···O3W^iii^	0.80 (2)	2.23 (2)	2.8310 (17)	132.3 (18)
N5—H1N5···N4^iv^	0.80 (2)	2.40 (2)	2.9925 (18)	131.2 (19)
N6—H1N6···O2W^ii^	0.83 (2)	2.47 (2)	3.209 (2)	147.6 (19)
N6—H2N6···O3^v^	0.93 (2)	2.06 (2)	2.9699 (17)	168.7 (19)
N7—H1N7···O3W^iii^	0.81 (2)	2.15 (2)	2.8570 (19)	145 (2)
N7—H2N7···O7^vi^	0.82 (2)	1.96 (2)	2.7714 (18)	170 (2)
O1W—H1W1···O2	0.79 (2)	2.03 (3)	2.7985 (19)	168 (3)
O1W—H2W1···O5^v^	0.77 (3)	2.11 (3)	2.8715 (17)	171 (2)
O2W—H1W2···O1W	0.90 (3)	1.81 (3)	2.703 (2)	173 (3)
O2W—H2W2···O1^i^	0.85 (3)	2.58 (3)	3.3968 (18)	162 (3)
O3W—H1W3···O6^vii^	0.89 (3)	1.90 (3)	2.7712 (17)	169 (2)
O3W—H2W3···O3	0.77 (3)	2.04 (3)	2.8113 (16)	177 (2)
C5—H5A···O6^viii^	0.93	2.35	3.2042 (18)	153
C12—H12A···O7^i^	0.93	2.47	3.3960 (17)	176

Symmetry codes: (i) *x*+1/2, *y*, −*z*+1/2; (ii) −*x*+3/2, *y*+1/2, *z*; (iii) *x*+1/2, −*y*+1/2, −*z*; (iv) −*x*+1, −*y*+1, −*z*; (v) −*x*+1/2, *y*+1/2, *z*; (vi) *x*+1, *y*, *z*; (vii) *x*−1/2, −*y*+1/2, −*z*; (viii) −*x*+1, −*y*, −*z*.
